# Effects of Phenolic Evolution on Color Characteristics of Single-Cultivar *Vitis vinifera* L. Marselan and Merlot Wines during Vinification and Aging

**DOI:** 10.3390/foods13030494

**Published:** 2024-02-03

**Authors:** Hua-Lin Zhang, Nong-Yu Xia, Xue-Chen Yao, Chang-Qing Duan, Qiu-Hong Pan

**Affiliations:** 1 Center for Viticulture and Enology, College of Food Science and Nutritional Engineering, China Agricultural University, Beijing 100083, China; zhldgzyx@foxmail.com (H.-L.Z.); xiany@cau.edu.cn (N.-Y.X.); yaoxch@cau.edu.cn (X.-C.Y.); chqduan@cau.edu.cn (C.-Q.D.); 2 Key Laboratory of Viticulture and Enology, Ministry of Agriculture and Rural Affairs, Beijing 100083, China

**Keywords:** red wine, anthocyanins, polymerized pigments, color stability, partial least squares regression analysis

## Abstract

The loss of red hue in dry red wine has been a persistent issue for wine enterprises in western China. We investigated the changes in anthocyanins and non-anthocyanin phenols during the industrial-scale fermentation and one-year bottle aging of *Vitis vinifera* L. Merlot and *Vitis vinifera* L. Marselan, respectively, using the grapes in the Ningxia region. We also examined their correlation with color characterization. The study found that both anthocyanins and non-anthocyanin phenolics were rapidly extracted from grapes during alcohol fermentation. However, their concentrations decreased rapidly during malolactic fermentation. On the other hand, Vitisin A and Vitisin B were formed during alcoholic fermentation and decreased slowly from malolactic fermentation to storage period. Directly polymerized pigments (F-A and A-F), bridged polymerized pigments (A-e-F), and flavanyl-pyranoanthocyanins (A-v-F) from the reactions of anthocyanins (A) and flavan*-3-*ols (F), as well as pinotins were generated during the later stages of alcoholic fermentation, and remained at a high level throughout malolactic fermentation and bottle storage. Partial least squares regression and Pearson correlation analyses revealed that the red hue (a* value) of ‘Merlot’ and ‘Marselan’ wines was closely associated with monomeric anthocyanins and F-A type pigments. Furthermore, four pinotin components were positively correlated with the red hue (a* value) of ‘Merlot’ wine. These primary red components of the two varieties had a positive correlation with the level of flavan*-3-*ols. The data suggest that elevating the flavan*-3-*ol concentration during fermentation aids in improving the color stability of red wine.

## 1. Introduction

Grape-derived phenolics consist mainly of anthocyanins, flavonols, flavan*-3-*ols, and phenolic acids. These compounds play a crucial role in determining the color, taste, aging potential, and health benefits of red wine. The type and concentration of anthocyanins determine the color depth and hue characteristics of wine. These compounds are leached from the grapes and evolve during wine-making and aging. Research has demonstrated that various factors, including temperature [1], light [2], and harvest time [3] can have a significant impact on the accumulation of anthocyanins in grape berries. Furthermore, vinification techniques can alter the levels and types of anthocyanins present in wine [4,5,6]. The monomers and acylated anthocyanins derived from grapes are highly unstable, and their concentrations decrease due to oxidation, degradation, or transformation into anthocyanin derivatives during cold soaking and alcohol fermentation [7]. This results in the evolution of red wine toward a yellow hue. The stability of anthocyanins is crucial for the color stability of wine during shelf life and storage.

Non-anthocyanin phenols in wine can induce intermolecular co-pigmentation, which enhances color and causes the red-shift effect. These phenols also affect the taste and aging potential of wine. Flavonols exist solely in glycosidic form in grape berries, while both free and glycosidic forms exist in wine. Flavonols have a light-yellow hue and can interact with anthocyanins in wine, helping to preserve the wine’s vibrant red color [7]. At a pH of 3.6, rutin binds with anthocyanins, resulting in a 16 nm red-shift effect and increased thermal stability of the anthocyanins [8]. Flavan*-3-*ols are found in grape skins and seeds, as free monomers and polymeric tannins. They can form red polymeric pigments with anthocyanins, which play a crucial role in maintaining the color stability of wine [9,10,11]. Phenolic acids, which are non-flavonoid compounds, are instrumental in the post-harvest handling, storage, and preservation of grapes. They also help to maintain the flavor, astringency, and nutritional value of wine. Previous research studies, both in simulated and actual wine systems, have confirmed that the addition of gallic acid [12], ferulic acid [8], and caffeic acid [13], along with flavonols, flavanols, and oak tannins, significantly influence the stability and color presentation of anthocyanins. Therefore, it is crucial to understand the leaching and evolution patterns of non-anthocyanin phenols and anthocyanins from grapes during fermentation and storage to maintain the color quality of single-variety wines and regulate the color in blended wines of multiple varieties.

Several research studies have shown that using cold maceration prior to fermentation can enhance the extraction of pigments and tannins from grapes, leading to a better color and quality of wine [14,15,16,17]. As wine ferments, alcohol content rises, which also improves the internal diffusion and mass transfer coefficients, making it easier to extract anthocyanins [18]. Anthocyanin molecules can bind to small molecules such as pyruvate, acetaldehyde, and acetone, as well as to substances with hydroxycinnamic acid structures, to form pyran anthocyanins [19,20]. Additionally, they can form polymeric pigments with flavanol molecules either directly or through acetaldehyde linkage, or even with other anthocyanin molecules [21,22]. Compared to monomeric and acylated anthocyanins from grapes, the anthocyanin derivatives produced through fermentation processes demonstrate superior stability. Some of these derivations can resist SO_2_ bleaching and pH changes to varying extents. During our investigation on the leaching and evolution of phenolic compounds in Cabernet Sauvignon wine fermentation with whole-process skin–seed contact, we observed a rise in polymeric pigments during the malolactic fermentation phase. This is accompanied by a notable decline in grape-derived anthocyanins [23]. However, variations in the phenolic profile among different varieties may result in differences in the types and patterns of anthocyanin derivatives produced during fermentation and aging processes.

Xinjiang and Ningxia are the largest wine-producing regions in China, accounting for 65% to 70% of the country’s annual wine production. Both regions have a semi-arid temperate climate, falling under the continental climate category. The annual rainfall is approximately 200 mm, while the evaporation rate ranges between 800 and 1000 mm. The temperature variation between day and night is between 15 and 20 °C, and the region enjoys over 3000 h of sunlight annually. Extreme temperatures exceeding 35 °C frequently occur [24]. The climatic conditions in this region cause the grapes to mature quickly, resulting in significant differences in the accumulation of anthocyanins, flavanols, tannins, and flavonols compared to grapes of the same variety from eastern China. As a result, the wine loses its red color and develops a yellow hue more quickly [25].

This study examines the leaching and evolution of various phenolic compounds and their impact on wine coloration throughout a one-year period, from ripe grape berries to wine fermentation and bottle storage, under local industrial-scale production conditions. Two red wine-making grape varieties *Vitis vinifera* L. ‘Merlot’ and ‘Marselan’ used in this study are mainly cultivated in the eastern foothills of the Helan Mountains in Ningxia. The objective of this investigation is to identify the key components responsible for the red coloration and establish a theoretical foundation for the targeted regulation of their leaching and accumulation. This will aid in the technological regulation of wine’s red hue.

## 2. Materials and Methods

### 2.1. Chemicals

Analytical grade chemicals, including sodium hydroxide, methanol, hydrochloric acid, sodium acetate, acetone and phloroglucinol, were purchased from the Beijing chemical plant (Beijing, China). Chromatographic-grade solvents, including methanol and acetonitrile, were acquired from Honeywell (Marris Township, NJ, USA), and formic acid (≥99%) was purchased from ROE Scientific (Newark, NJ, USA). The authentic standards of malvidin*-3-O-*glucoside, quercetin*-3-O-*glucoside, catechin, (−)-epicatechin, (−)-epigallocatechin, and (−)-epicatechin*-3-O-*gallate were obtained from Sigma-Aldrich (St. Louis, MO, USA).

### 2.2. Winemaking and Sampling

**Figure 1:**
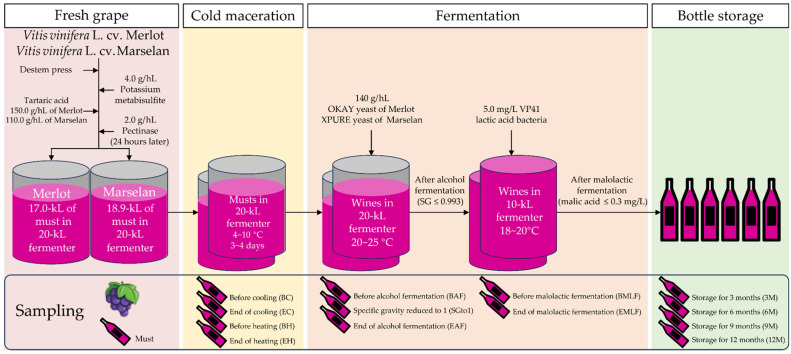
Schematic diagram of winemaking and sampling.

In 2021, an experiment was conducted at Chateau Zhihui Yuanshi (38°28′ N, 106°12′ E), located in the eastern foothills of the Helan Mountains, Ningxia, China. *V. vinifera* L. cv. Marselan and *V. vinifera* L. cv. Merlot were harvested from the vineyards. The grapes underwent destemming and sorting on an LXZDS-5 sorting table obtained from Leading Machinery, Xinxiang, China, to remove any diseased or unripe berries. Approximately 17,000 kg of ‘Merlot’ and 18,900 kg of ‘Marselan’ grapes, respectively, were gently crushed and added to a 20-kiloliter (kL) fermenter along with 4.0 g/hL of K₂S₂O_5_ and 2.0 g/hL of pectinase. To compensate for the low titratable acid content in the grapes, 150 g/hL of tartaric acid was added for ‘Merlot ‘and 110 g/hL for ‘Marselan’. The mixture was then cold macerated at 4–10 °C for 3–4 days. At a temperature of 20 °C, 14.0 g/hL of OKAY yeast (LALLEMAND, Niagara-on-the-Lake, ON, Canada) and XPURE yeast (Laffort, Bordeaux, France) were added for ‘Merlot’ and ‘Marselan’, respectively, to initiate alcohol fermentation. The fermentation process was carried out at a temperature range of 20–25 °C, with the wine being pumped every 8 h for 20 min from the beginning of cold maceration until the end of alcohol fermentation. Throughout alcohol fermentation, the temperature and specific gravity of the wine were closely monitored. When the specific gravity reached about 0.993, the alcohol fermentation process was considered complete. The wine was then transferred to a 10-kL fermenter after racking, to initiate malolactic fermentation by using 5.0 mg/L VP41 lactic acid bacteria (Enartis, Niagara-on-the-Lake, ON, Canada). The temperature was controlled at 18–20°C. After the malolactic fermentation was complete, indicated by a malic acid level falling below 0.3 mg/L, the sulfur dioxide concentration in the wine was adjusted to 20 mg/L. Then, the wine was bottled for storage in a cellar with a constant temperature of 16–18 °C and a relative air humidity of 60–70% (Figure 1).

At 8 a.m. on the day of harvest, mature grape berries were collected. Three plots were selected from the ‘Marselan’ and ‘Merlot’ vineyards as three biological replicates. From each plot, 200 berries were randomly selected from each plot, frozen with liquid nitrogen, and stored in a −80 °C refrigerator for analysis. Sampling was carried out at the stages of cold maceration, alcohol fermentation, malolactic fermentation, and storage. Three bottles of 350 mL were taken for each variety at each sampling event. The specific dates of sampling are given in Table S1. The abbreviations and their full names in this article are given in the Abbreviations.

### 2.3. Measurement of Physicochemical Parameters

For each grape sample, we weighed 50 berries and extracted their juice by squeezing. We measured the total soluble solid (TSS) of the juice using a pocket Brix refractometer (PAL-1; ATAGO, Tokyo, Japan), and determined the pH value using a calibrated pH meter (FE20; Mettler Toledo, Greifensee, Switzerland). To calculate the titratable acid content, we titrated 0.1 mol/L NaOH with phenolphthalein as an indicator (expressed as tartaric acid, g/L) [26].

Wine samples used for physicochemical parameters were collected at the end of malolactic fermentation. Residual sugar, total acid, volatile acid (VA), and pH value were measured using a Foss Winescan (Foss Electric, Hillerød, Denmark) fast-scanning infrared Fourier-transform spectrometer.

### 2.4. Measurement of Chromatic Parameters

The 2 mL wine sample was filtered using a 0.45 μm polyethersulfone filter (Jinteng Experimental Equipment Co., Ltd., Tianjin, China), and the filter was added to a 2 mm path-length quartz cuvette. The wine’s CIELab parameters were analyzed on a CM-3700A spectrophotometer (Konica Minolta, Tokyo, Japan) with distilled water as a reference. The parameters analyzed were brightness (L*), red/green value (a*), and yellow/blue value (b*). The chroma saturation (C*_ab_), hue angle (*h_ab_*), and color difference (ΔE*_ab_) were calculated using Equations (1)–(3) [27]:

C*_ab_ = ((Δa*)^2^ + (Δb*)^2^)^1/2^, (1)

*h_ab_* = tan^−1^ (b*/a*), (2)

ΔE*_ab_ = ((ΔL*)^2^ + (Δa*)^2^ + (Δb*)^2^)^1/2^, (3)

### 2.5. Extraction of Phenols from Grape Skins

Anthocyanins and flavonols were extracted from grape skins following the method previously reported [28]. In brief, 0.100 ± 0.002 g of lyophilized grape skin powder was mixed with 1 mL of a 50% (*v*/*v*) methanol aqueous solution. The mixture underwent ultrasonic extraction at 20 °C for 20 min, followed by centrifugation at 8000 r/min for 10 min, and the supernatant was collected. The residue was extracted twice using the same procedure.

Free flavan*-3-*ol monomers were extracted using the method of Wang et al. [29]. Specifically, 0.100 ± 0.002 g of lyophilized grape skin powder was mixed with 1 mL of 70% acetone aqueous solution (containing 0.5% ascorbic acid), and the mixture was sonicated for 20 min. The supernatant was collected by centrifugation, and the residue was re-extracted three times. Finally, 400 μL of the supernatant was dried under nitrogen in the dark. The dried extract was dissolved again with 200 μL of methanol containing 1% HCl. Then, 200 μL of aqueous sodium acetate (200 mM) was added for neutralization. The flavan*-3-*ol content was measured using the neutralized extract.

Flavan*-3-*ol polymers (proanthocyanidin, PA) were extracted according to our previous report [29]. A total of 0.050 g of lyophilized grape skin powder was mixed with 0.5 mL of phloroglucinol solution that composed of 0.3 mol/L of HCl, 50 g/L of phloroglucinol, and 0.5% of ascorbic acid. The mixture was incubated in a 50 °C water bath for 20 min, and the reaction was terminated by adding 0.5 mL of 200 mM aqueous sodium acetate. The supernatant was collected by centrifugation, and the procedure was repeated three times before pooling the supernatant.

### 2.6. Analysis of Phenolic Compounds in Grape and Wine

Phenolic compounds in grapes and wine were tested using HPLC-QQQ-MS/MS, as previously described [30]. The grape extraction solution and wine were filtered through a 0.22 μm organic phase filter prior to analysis. The HPLC column used was Poroshell 120 EC-C18 (150 mm × 3.0 mm, 2.7 μm), with a 5 μL injection volume. The mobile phase flowed at a rate of 0.4 mL/min, and the column temperature was maintained at 55 °C. The mobile phase was composed of (A) a 0.1% aqueous solution of formic acid and (B) a mixture of methanol and acetonitrile (50/50, *v*/*v*) containing 0.1% formic acid. A gradient elution procedure was employed: 0–28 min, 90% to 54% solvent A and 10% to 46% solvent B; 28–29 min, 54% to 90% solvent A and 46% to 10% solvent B; held for 5 min after the running time. The mass spectrometer utilized an electrospray ionization (ESI) source to detect anthocyanins in positive ion mode and non-anthocyanin phenols in negative ion mode. The ion source conditions were set to a spray voltage of 4 kV, temperature of 150 °C, dry gas temperature of 350 °C, flow rate of 12 L/h, and atomizer pressure of 35 psi. The mass spectrometric information of target compounds was collected under multi-reaction monitoring mode.

Phenolic compounds were identified and quantified using a self-developed library of phenolic compounds and the external standard method [31]. Anthocyanins and flavonols were quantified using malvidin*-3-O-*glucoside and quercetin*-3-*glucoside as the external standards, respectively. Flavan*-3-*ol components were quantified using (*+*)-catechin, (−)-epicatechin, (−)-epigallocatechin, and (−)-epicatechin*-3-O-*gallate as the external standards. Phenolic concentration was measured in mg/kg based on the fresh weight of the fruit.

### 2.7. Statistical Analysis

A one-way ANOVA (Duncan, *p* < 0.05) was performed using the ‘agricolae’ package (https://CRAN.R-project.org/package=agricolae (accessed on 22 October 2023)) in R software (version 4.1.3). The hierarchical clustering in the heatmap was created using the ‘pheatmap’ function within the ‘pheatmap’ package (https://CRAN.R-project.org/package=pheatmap (accessed on 1 October 2023)). Correlation analysis was carried out using the ‘corrplot’ function within the ‘corrplot’ package (https://CRAN.R-project.org/package=corrplot (accessed on 18 November 2023)). The PLSR model was analyzed using the ‘pls’ package (https://CRAN.R-project.org/package=pls (accessed on 17 November 2023)) in R software. All images were created using the ‘ggplot2’ package (https://CRAN.R-project.org/package=ggplot2 (accessed on 12 October 2023)) in R software. The color palette was created by importing the measured L*, a*, and b* values into Adobe Photoshop CS2018 software (Adobe Systems, Inc., San Jose, CA, USA).

## 3. Results

### 3.1. Phenolic Profiles of the Grapes of Two Varieties

#### 3.1.1. Anthocyanins

Fifteen anthocyanin components were identified in the grape skins of the two varieties. These components were in the form of glucoside and the acylated glucoside of cyanidin, peonidin, delphinidin, petunidin, and malvidin. In the ‘Merlot’ grape skins, cyanidin- and peonidin-type anthocyanins with two substituents on the B ring, except for peonidin*-3-O-*acetylglucoside, were found to be present in higher levels than in the ‘Marselan’ grape skins. However, the opposite pattern was displayed by anthocyanins with three substituents on the B ring. Except for petunidin*-3-O-*acetylglucoside, the other eight components showed significantly lower levels in ‘Merlot’. It is noteworthy that the concentrations of three malvidin-type anthocyanins, which are abundant in grape skins, were less than half the amount found in the ‘Marselan’ grape skins. According to Table 1, ‘Marselan’ contained 731.04 mg/kg of total anthocyanins, which is 1.5 times more than ‘Merlot’ with 481.92 mg/kg.

#### 3.1.2. Non-Anthocyanin Phenolics

Nine flavonol compounds were identified in the grapes of ‘Merlot’ and ‘Marselan’. In ‘Marselan’, most compounds, such as kaempferol, quercetin, and isorhamnetin, with one or two substituents on the B ring, had higher concentrations than those in ‘Merlot’, while myricetin and syringetin with three substituents had lower concentrations than those in ‘Merlot’ (Table 1). Overall, the concentration of flavonols in ‘Marselan’ (212.26 mg/kg) was higher than that in ‘Merlot’ (181.79 mg/kg).

The ‘Marselan’ grape skins showed a higher level of only epicatechin monomer compared to ‘Merlot’. However, four proanthocyanidin components were identified, including catechin, epicatechin, epigallocatechin, and epicatechin gallate. The concentrations of extension units were significantly higher in the ‘Marselan’ grape skins than in ‘Merlot’, while the concentrations of end units were notably lower. As a result, the mean degree of polymerization of ‘Marselan’ (32.19) was significantly higher than that of ‘Merlot’ (13.05) (Table 1).

### 3.2. Physicochemical Indices and Chromaticity Index

#### 3.2.1. Physicochemical Indices

Both grape varieties had total soluble solids exceeding 25.0 °Brix, with consistently low titratable acids, indicating the ‘high sugar and low acid’ characteristic found in grapes from the eastern foothills of Ningxia’s Helan Mountains. This characteristic results in wines with high alcohol concentrations (Table 2). All other parameters met the quality standards for dry red wine in GB/T 15037-2006 Wine [26]. It is important to note that the titratable acid content of the wines exceeded that of the grape juice. This was mainly due to the addition of tartaric acid before alcohol fermentation, as per the local production guidelines. A specific amount of tartaric acid was added to adjust the pH of the wine and improve its color stability and taste structure to a certain extent.

#### 3.2.2. Chromaticity Index

**Figure 2:**
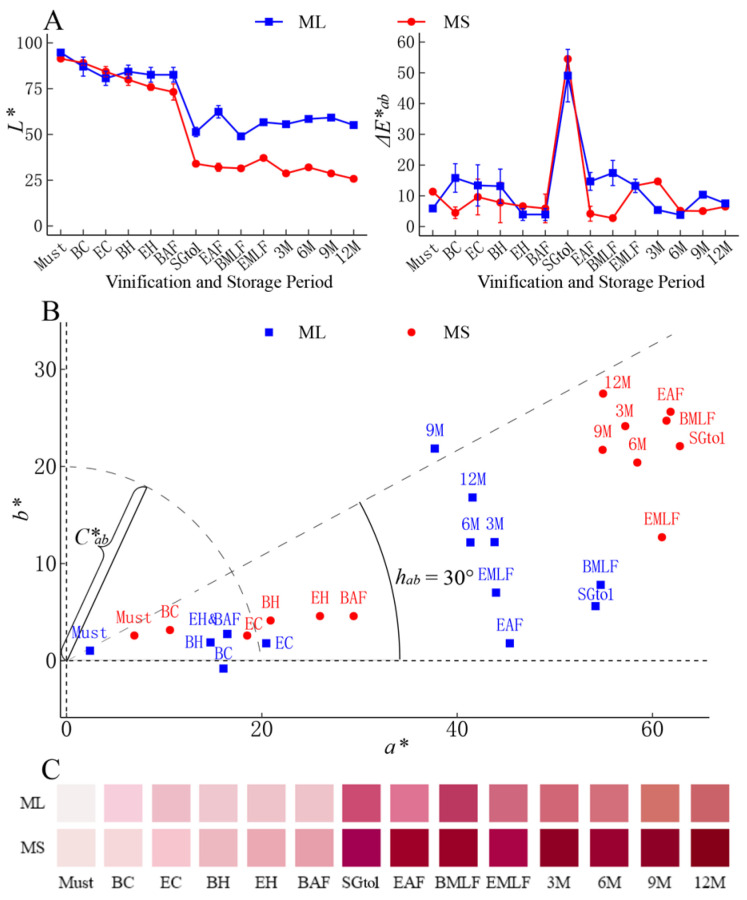
Variation in chromaticity index (**A**,**B**) and color (**C**) of ‘Merlot’ and ‘Marselan’ wines during fermentation and bottle storage. The descriptions of each period on the horizontal axis are given in Table S1 (the same below).

Figure 2A shows that the brightness L* value gradually decreases during the cold soak phase and sharply at the onset of alcoholic fermentation, and then stabilizes at a certain level after the specific gravity (SG) reaches 1.0. This indicates a deepening of color. The varietal difference was mainly observed in the stage following the initiation of alcoholic fermentation, with the L* value for ‘Marselan’ notably lower than that of ‘Merlot’. This result showed that ΔE*_ab_ had the highest value at a specific gravity of 1.0 for both the ‘Merlot’ and ‘Marselan’ wines, indicating the most rapid change in wine color during this period.

Figure 2B illustrates a progressive increase in the red hue a* value from the cold maceration to alcohol fermentation stages in the ‘Merlot’ and ‘Marselan’ wines. After the specific gravity fell to 1.0, the a* value decreased gradually. The Y-axis shows the gradual increase in the yellow hue b* value. During the cold maceration stage, both varieties had low b* values. As alcoholic fermentation began, significant differences emerged between the two varieties. Specifically, the b* value for ‘Merlot’ wine steadily increased, while ‘Marselan’ experienced a rapid rise, followed by a rapid decrease during the malolactic fermentation phase, and then rebounded during the storage phase. In this process, the b* value for ‘Marselan’ wine consistently exceeded that of ‘Merlot’. Throughout various stages, the a* and b* values for ‘Marselan’ wine were notably higher than those for ‘Merlot’.

The C*_ab_ represents the distance from the point to the base point. In this study, its trend is comparable to that of the a* value, highlighting that the C*_ab_ of the ‘Marselan’ wine was notably higher compared to that of the “Merlot” wine in the same period. Furthermore, it was observed that all the hue angle *h_ab_* points were distributed below the 30° line, indicating that red color was the primary hue in both wine varieties. The ‘Marselan’ wine shifted towards a red hue during cold maceration and alcohol fermentation, and then transitioned at a slower pace to a yellow hue from the end of fermentation to a year of storage. The variation trend of *h_ab_* in ‘Merlot’ wine was similar to that of ‘Marselan’ wine during cold maceration. However, the “Merlot” wine experienced a more pronounced increase in *h_ab_* following the end of the alcoholic fermentation. This suggests a quicker evolution towards a yellow hue during bottle storage.

The colors of both ‘Merlot’ and ‘Marselan’ wines were simulated (Figure 2C). Anthocyanins in the grape skins were gradually leached with the progression of cold maceration and fermentation, resulting in a complete color change to dark purple. Compared to the ‘Merlot’ wine, the color of the ‘Marselan’ wine appeared deeper and ruddier at various stages.

### 3.3. Phenolic Profiles of the Wines of Two Varieties

#### 3.3.1. Anthocyanins and Their Derivatives in Wine

Table 3 shows the concentrations of different phenolic compounds categorized by structure in the ‘Merlot’ and ‘Marselan’ wines after malolactic fermentation. The wines contained 15 grape-derived anthocyanins, 10 pyranoanthocyanins, and 6 polymeric pigments in the case of ‘Merlot’ wine and 8 in the case of ‘Marselan’ wine. The ‘Merlot’ wine had higher levels of cyaniding-type and peonidin-type anthocyanins and lower levels of delphinidin-type anthocyanins in comparison to the ‘Marselan’ wine, where the trends were similar to those in the berries. However, there were no significant differences in the concentrations of petunidin-type and malvidin-type anthocyanins between the wines of the two varieties, which differed from the trends observed in the berries. This suggests that these two types of anthocyanins were more susceptible to degradation or transformation into anthocyanin derivatives during fermentation.

This study identified pyranoanthocyanins, specifically, Vitisin A, Vitisin B, and pinotins. These compounds are formed through the cyclization of pyruvic acid, acetaldehyde, and cinnamic acid, with the C4 and C5 sites on the three forms of malvidin-type anthocyanins [32]. Pinotins are characterized by an orange-red color [33]. In this study, four types of pinotin were detected: Mv-vpol, Mv-vgol, MvA-vpol, and MvC-vphol. The ‘Merlot’ wine had higher levels of Vitisin B and pinotin, and lower levels of Vitisin A compared to the ‘Marselan’ wine.

Polymeric pigments found in wine can be classified into direct- and bridge-linked types, which typically exhibit a red hue [33,34]. The ‘Marselan’ wine contained five types of flavanol-anthocyanin (F-A) or A-F polymeric pigments, namely Mv-(e)cat, (E)cat-Mv, (E)cat-MvA, and (E)cat-MvC. However, the ‘Merlot’ wine only contained (E)cat-Mv and (E)cat-MvA. Additionally, both wines contained four types of bridge-linked polymeric pigments (A-e-F) which were identified as Mv-e-(e)cat, MvA-e-(e)cat, MvC-e-(e)cat, and Mv-e-di(e)cat. The concentration of F-A (A-F) type pigments was higher in comparison to the ‘Merlot’ wine, while no significant difference was observed in A-e-F type pigments (Table 3).

#### 3.3.2. Non-Anthocyanin Phenolics in Wine

Both wines contained 10 flavonols and 5 flavan*-3-*ols. The ‘Merlot’ wine had nine phenolic acids, while the ‘Marselan’ wine had eleven. The ‘Merlot’ wine exhibited higher levels of these compounds compared to the ‘Marselan’ wine (Table 3).

### 3.4. Leaching and Evolution of Phenolics from Grape to Wine

**Figure 3:**
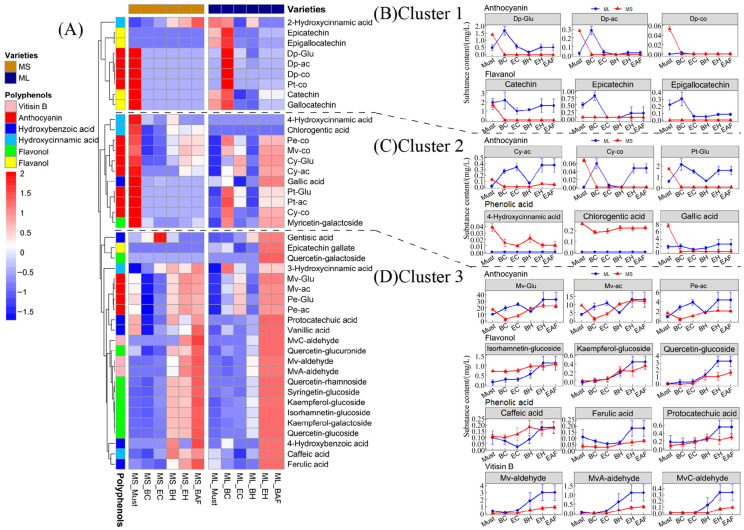
Evolution of phenolic compounds during cold maceration before fermentation. (**A**) Clustered heat map of the concentrations of phenolic compounds. (**B**) The changes in the concentration of representative four anthocyanins and four flavan*-3-*ols in Cluster 1. (**C**) The changes in the concentration of representative four anthocyanins and three phenolic acids in Cluster 2. (**D**) The changes in the concentration of representative four anthocyanins, three Vitisin B, and non-anthocyanin phenols in Cluster 3.

**Figure 4:**
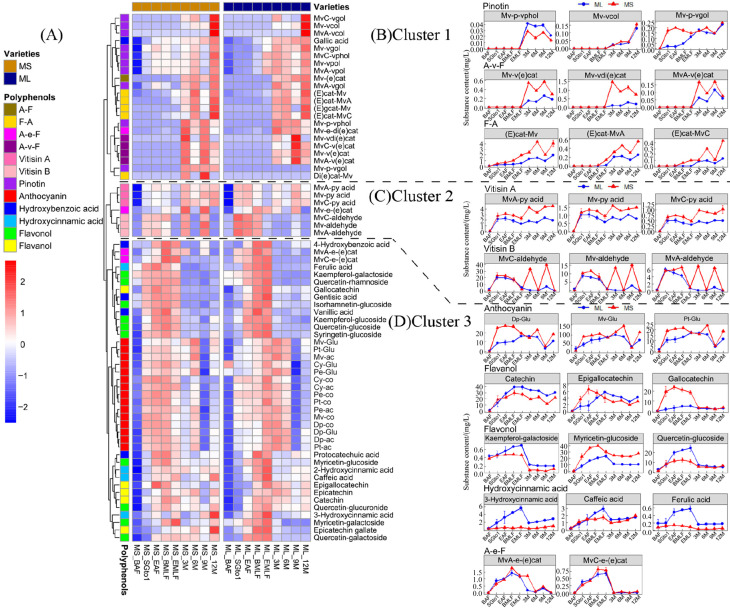
Variation trend of phenolic compounds in the process of fermentation and one-year bottle storage. (**A**) Clustered heat map of the concentrations of phenolic compounds. (**B**) The changes in the concentration of representative pinotin, F-A, and A-v-F in Cluster 1. (**C**) The changes in the concentration of representative Vitisin A and Vitisin B in Cluster 2. (**D**) The changes in the concentration of representative anthocyanins, polymeric pigments A-e-F, and some non-anthocyanin phenols in Cluster 3.

During the vinification process, grape-derived phenolic compounds typically undergo continuous leaching and transformation from the water phase (refer to the per-fermentation cold maceration stage) to the alcohol phase (refer to the fermentation and bottle storage stages). To better illustrate the leaching and evolutionary patterns of phenolics, we conducted hierarchical cluster analyses for the water (Figure 3) and alcohol phases (Figure 4), respectively. Additionally, Tables S2 and S3 display the concentrations of phenolic compounds during the fermentation and bottle storage processes of the two wine varieties, excluding the compounds shown in the line graphs of Figure 3 and Figure 4.

#### 3.4.1. Leaching of Phenolics during Cold Maceration

During the cold maceration process, phenolic compounds were clustered into three groups based on their variation trends (Figure 3A). No leaching was observed for the components in Cluster 1, which primarily consisted of three delphinidin-type anthocyanins, petunidin*-3-O-*glucoside, and four flavan*-3-*ol compounds (Figure 3B). However, high concentrations of certain components were observed at the first sampling point of ‘Marselan’ and the first two sampling points of ‘Merlot’. This may be due to the absence of juices at that time, which required us to centrifuge the must to obtain the supernatants. This process caused a forced leaching of compounds from the grape skins. For the samples from other sampling points, we collected juice directly from the must for filtration and analysis.

Cluster 2 contained three phenolic acids, seven anthocyanins, and one flavonol. These compounds showed significant varietal differences during leaching. For the ‘Merlot’ wine, all compounds in this cluster, except for 4-hydroxycinnamic acid and chlorogenic acid, presented a slow increase in concentration. However, the leaching of most compounds was very limited for the ‘Marselan’ wine (Figure 3C).

Cluster 3 comprised four anthocyanins, three Vitisin B, and the majority of flavonols and phenolic acids. The two varieties exhibited an increasing trend in the compound concentrations during cold maceration (Figure 3D). Although, of those, the leaching of phenolic compounds, especially flavan*-3-*ols, was limited during the cold maceration stage.

#### 3.4.2. Evolution of Phenolics during Fermentation and Bottle Storage

During the process of alcoholic fermentation, malolactic fermentation, and one-year bottle storage, the changes in phenolic compounds were grouped into three clusters (Figure 4A). Cluster 1 compounds did not significantly increase or were not generated during alcoholic fermentation and malolactic fermentation, but their concentrations rapidly increased during the bottle storage stage. The compounds found in the wines were primarily pyranoanthocyanins, F-A type polymeric pigments, and flavanyl-pyranoanthocyanins (A-v-F). Pinotins (e.g., Mv-vpol) were found in higher concentrations in the ‘Merlot’ wine stored in bottles compared to the ‘Marselan’ wine. On the other hand, F-A and A-v-F compounds were more abundant in the ‘Marselan’ wine stored in bottles (as illustrated in Figure 4B). During the bottle storage phase, both wine varieties showed an increase in the concentration of the A-F type polymeric pigment, Mv-(e)cat.

Additionally, the compounds in Cluster 2 accumulated rapidly during alcohol fermentation, followed by a slight decline during malolactic fermentation. These compounds were primarily Vitisin A and Vitisin B, which are related to the generation of their substrates, namely acetaldehyde and pyruvate. During bottle storage, the concentration of Vitisin A remained high in both varieties, with a higher concentration in the ‘Marselan’ wine than in the ‘Merlot’ wine. However, the concentration of Vitisin B dropped sharply to a very low level in the ‘Merlot’ wine and fluctuated substantially in the ‘Marselan’ wine (Figure 4C).

The compounds in Cluster 3 experienced a rapid surge during the alcohol fermentation phase and reached a peak shortly thereafter. There was a decrease of a varying degree during the malolactic fermentation and subsequent bottle storage. The compounds included grape-derived anthocyanins, A-e-F type polymeric pigments, as well as a majority of flavan*-3-*ols, flavonols, and hydroxycinnamic acid. During bottle storage, two A-e-F polymeric pigments, MvA-e-(e)cat and MvA-e-(e)cat, significantly dropped to a very low level (Figure 4D).

### 3.5. Key Components for Wine Color Expression

**Figure 5:**
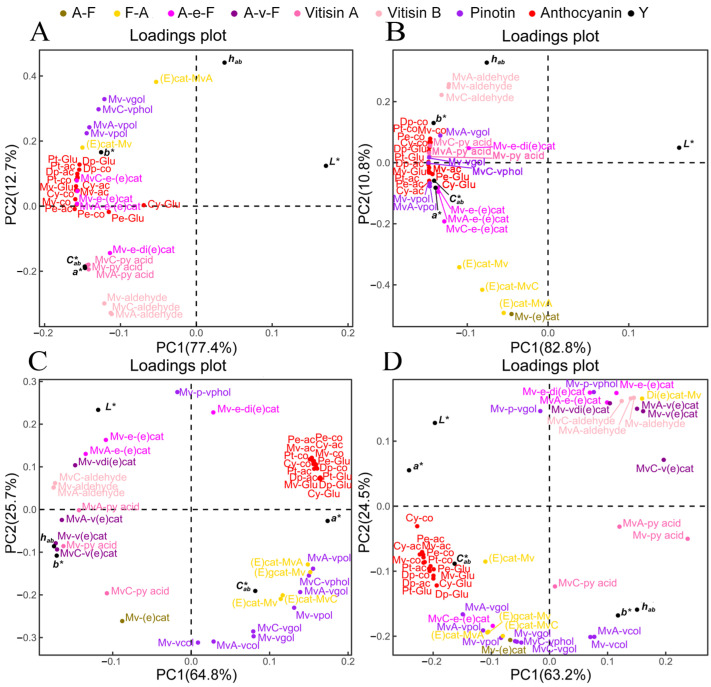
Partial least squares regression (PLSR) analysis based on CIELAB parameters and the concentrations of anthocyanins and their derivatives for ‘Merlot’ (**A**) and ‘Marselan’ (**B**) wines during vinification, and for ‘Merlot’ (**C**) and ‘Marselan’ (**D**) wines during bottle storage.

To demonstrate the influence of phenolic compounds on wine color during vinification, we performed a partial least squares regression (PLSR) analysis. We used the concentrations of anthocyanins and their derivatives in wine during fermentation and bottle storage as independent variables (X), and the CIELab color parameters as dependent variables (Y). The correlation model was established, and the R^2^X, R^2^Y, and Q^2^ values were 0.971, 0.739, and 0.531 for the ‘Merlot’ wine and 0.985, 0.992, and 0.987 for the ‘Marselan’ wine, respectively. These results indicate strong discriminant analysis capabilities. The L* values of both the ‘Merlot’ (Figure 5A) and ‘Marselan’ (Figure 5B) wines were observed to be in the region of the X positive axis, while the detected grape-derived anthocyanins and their derivatives were all in the region of the negative X axis. This indicates a negative correlation between the anthocyanin compounds and the L* value, and a strong positive correlation between the anthocyanin compounds and the a*, b*, and C*_ab_ values. Additionally, the values of a*, b*, and C*_ab_ values showed a strong correlation with each other in the fourth quadrant. The main distinction between the two varieties was the *h_ab_* value. Throughout the fermentation of ‘Merlot’ wine, the *h_ab_* exhibited a negative correlation with a*, b*, and C*_ab_ values. This suggests that an increase in these compounds could result in a greater increase in the red hue than the yellow hue (Figure 5A). In contrast, the *h_ab_* showed a positive correlation with these three chromaticity indexes while fermenting ‘Marselan’ wine (Figure 5B). This suggests that the changes in these compounds could cause a synchronous enhancement of red and yellow hues, without dramatically altering the overall hue.

PLSR analysis was also conducted for the data from the bottle storage stage. The analysis revealed a strong discriminant capability based on the R^2^X, R^2^Y, and Q^2^ values of 0.991, 0.991, and 0.980 for the ‘Merlot’ wine, and 0.983, 0.995, and 0.988 for the ‘Marselan’ wine. It was observed that the *h_ab_* and b* values had a close correlation with each other in the two wine varieties. In the ‘Merlot’ wine (Figure 5C), the *h_ab_* and b* values showed a positive correlation with all Vitisin A, four A-v-F polymeric pigments, and three Vitisin B components. This suggests that the evolution of these compounds significantly affected the yellow hue of the wine during the bottle aging process. Concurrently, the values of a* and C*_ab_ were closely associated with the changes in grape-derived anthocyanins, four F-A type polymeric pigments, and four pinotins (Mv-vpol, MvA-vpol, MvA-vgol, MvC-vphol) located in the region of the X positive axis. A decline in both the red hue (Figure 2) and anthocyanin concentrations (Figure 4) was observed during the bottle storage process. The data suggest that both anthocyanins derived from grapes and F-A type polymeric pigments may contribute to the red hue during bottle storage of ‘Merlot wine’. Additionally, ‘Merlot’ wine showed higher levels of pinotins during the bottle storage phase compared to the ‘Marselan’ wine (Figure 4), indicating a correlation between the compounds and variations in the red hue.

Regarding the ‘Marselan’ wine, the yellow hue was found to be related to the evolution of three Vitisin A components (Mv-py acid, MvA-py acid, MvC-py acid) (Figure 5D). Besides, seven pinotin-type compounds (Mv-vpol, Mv-vcol, Mv-vgol, MvA-vpol, MvA-vcol, MvC-vphol, MvC-vgol) were found to be closely associated with *h_ab_* and b* values in the third quadrant, distinguishing it from the ‘Merlot’ wine. The grape-derived anthocyanins, F-A type polymeric pigments ((E)cat-Mv, (E)gcat-Mv, (E)cat-MvA, (E)cat-MvC), and A-F type polymeric pigment (Mv-(e)cat), as well as the a* value, were all positioned in the positive X axis region, indicating their contribution to the red hue (Figure 5D).

### 3.6. Correlation among Changes of Phenolic Compounds in Wine

**Figure 6:**
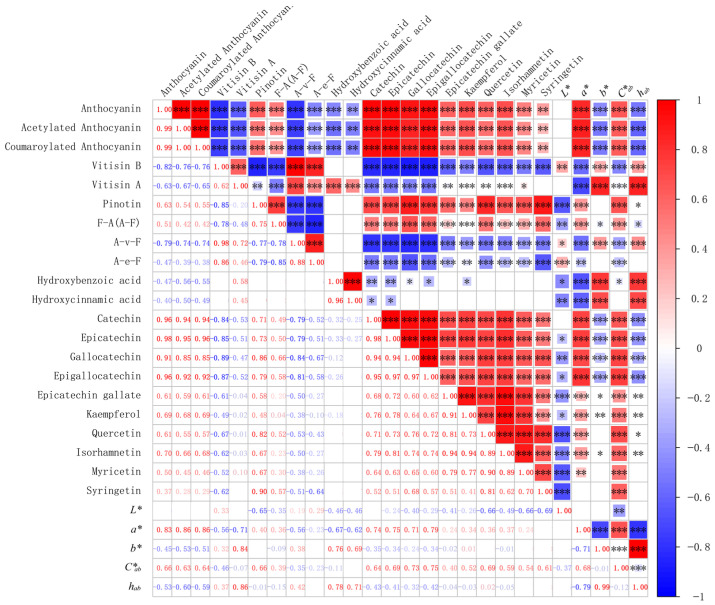
Correlation between the concentrations of phenolic compounds and chromaticity index in the ‘Merlot’ and ‘Marselan’ wines. The “*”, “**”, and “***” represented that the correlations were statistically significant, which means *p* < 0.05, *p* < 0.01, *p* < 0.001, respectively.

Non-anthocyanin phenolic compounds, specifically flavan*-3-*ols, contribute not only to co-pigmentation but also to the formation of polymeric pigments. To investigate the relationship between flavan*-3-*ols and the evolution of wine color components, we constructed a Pearson correlation model by using the chromaticity data and the concentrations of anthocyanins, and non-anthocyanin phenolic compounds in two wine varieties during storage. Figure 6 displays the correlation coefficient. A correlation coefficient greater than 0.5 or less than −0.5 is generally considered as a strong association [35].

This study found a strong negative correlation between the changes in catechin, epicatechin, gallocatechin, and epigallocatechin in the two wine varieties with A-v-F and A-e-F compounds as well as *h_ab_* and b* values. Additionally, a positive correlation was observed with F-A (A-F) compounds, pinotins, anthocyanins, and a* and C*_ab_ values. Epicatechin gallate showed a positive correlation with pinotins and anthocyanins, and a negative correlation with A-v-F. This suggests that the flavan*-3-*ols in wine decrease progressively during aging, accompanied with a degradation or transformation of F-A (A-F), pinotins, and anthocyanins. As the A-v-F and A-e-F levels gradually rose, the wine’s color became increasingly faded over aging, transitioning from a red hue to an orange-yellow one.

## 4. Discussion

This study analyzed the changes in concentrations of phenolic compounds in ‘Merlot’ and ‘Marselan’ wines from cold maceration and fermentation to bottle storage under industrial-scale production conditions. During the cold maceration phase, flavonols and phenolic acids, along with small amounts of anthocyanins, were found to leach from the grape skins. The number of anthocyanins was fewer in ‘Marselan’ than in ‘Merlot’, which may be due to the relatively thinner skins of ‘Merlot’ grapes, making them more susceptible to extraction. Phenolic compounds accumulated rapidly and peaked during alcohol fermentation, after which grape-derived phenolic compounds began to decline. This decrease may be caused by various factors, such as the adsorption of yeast distillers’ grains, enzymatic hydrolysis, oxidation, or the formation of copolymers from other non-anthocyanin phenols. These processes occur from the latter phase of alcoholic fermentation to the stage of malolactic fermentation and bottle storage [36]. Previous research has demonstrated that during this phase, anthocyanins combine with tannins or react with hydroxycinnamate to form more stable polymeric pigments [37,38].

Vitisin A exhibits a resistance to SO_2_ bleaching and robust antioxidant properties. It displays an orange-red hue in environments ranging from weakly acidic to nearly neutral pH [39]. In contrast, Vitisin B, the simplest pyrananthocyanin currently isolated from red wine, displays a distinct orange-yellow color in the visible spectrum [40]. In our study, we observed that Vitisin B formed at a very slow rate during cold maceration. However, both Vitisin A and Vitisin B experienced a rapid increase in concentration during alcohol fermentation, followed by a slight decrease during malolactic fermentation. The production of these two pigments plays a significant role in maintaining the stability of wine color during the fermentation period. During bottle storage, the level of Vitisin B decreased significantly in the ‘Merlot’ wine, while experiencing a significant fluctuation in the ‘Marselan’ wine. The difference in varietals is suggested to be related to the level of acetaldehyde in the wine. Previous studies have shown that the concentrations of Vitisins peak at the post-fermentation and early-aging stages, with Vitisin B levels being slightly lower than those of Vitisin A [41]. The color loss in wine following malolactic fermentation may be caused by several factors, such as the increase in pH during this process, the reduced ability of bacteria to produce pyruvate and acetaldehyde, and the adsorption of anthocyanins by bacterial cells [36]. The previous study also showed that malolactic fermentation resulted in lower levels of polymeric pigments and Vitisin A and B, and higher levels of grape-derived anthocyanins compared to wine without malolactic fermentation [36]. Pinotin has spectral characteristics similar to Vitisin, with a shift in the maximum absorption wavelength towards shorter wavelengths, indicating an orange-red hue [33]. In our study, it was observed that pinotins emerged during alcohol fermentation and then slightly increased, exhibiting notable accumulation at the beginning of bottle storage, maintained at a high level. Previous research has indicated that pinotin concentrations peak after 5–6 years of wine aging, which is approximately 10 times higher than that of fresh dry red wine during the initial aging phase [33].

During fermentation, F-A (A-F) and A-e-F pigments were formed and gradually increased. Their synthesis was accelerated during the bottle storage phase. These pigments primarily contribute to the red hue of aged wine. In the ‘Marselan’ wine, only a trace level of A-F pigment (Mv-(e)cat) was detected at the end of malolactic fermentation. However, in the ‘Merlot’ wine, this pigment was identified during bottle storage. During the fermentation phase for both varieties, F-A type polymeric pigments emerged and then rapidly accumulated during the aging period. The observed change is not only due to an increase in the concentration of existing compounds, but also the emergence of new compounds during the aging stage. For instance, (E)gcat-Mv was identified in both varieties, while Di(e)cat-MvA was exclusively observed in the ‘Marselan’ wine. However, it was observed that certain A-e-F pigments disappeared in the ‘Merlot’ wine, such as MvA-e-(e)cat and MvC-e-(e)cat. This disappearance may be attributed to the brittle ethylene in the molecular structure of these aldehyde-mediated bridged polymeric pigments [42]. These compounds exhibit low stability in wine [33]. Therefore, it is commonly believed that these pigments primarily exist in fresh wine and gradually degrade with aging [43]. A recent study observed that the degradation rate of this kind of anthocyanin derivatives during wine aging is significantly higher than that of their precursor anthocyanins [44].

According to the PLSR analysis, this study found that the red hue of ‘Merlot’ wine increased at a faster rate than the yellow hue during fermentation. In contrast, both hues increased concurrently in ‘Marselan wine’. Anthocyanins and F-A type polymeric pigments were identified as key components contributing to the red hue of both ‘Merlot’ and ‘Marselan’ wines during the bottle storage phase. Previous studies have shown that F-A type polymeric pigments have a maximum absorption wavelength of around 530–535 nm. For instance, (E)cat-Mv and Di(e)cat-Mv have absorption wavelengths of 529 nm and 530 nm, respectively [45]. This indicates a red shift effect when compared to Mv-Glu (520~525 nm) [46]. Therefore, it can be identified as a crucial component responsible for the purplish-red hue in this context. Moreover, pinotins display an orange-red hue [33], which is responsible for the red color of ‘Merlot’ wine and is consistent with its color evolution during aging.

The correlation analysis of phenolic compounds indicates a positive correlation between the decrease in the concentrations of flavan*-3-*ol compounds and the decrease in a* and C*_ab_ values during bottle storage, as well as a negative correlation with *h_ab_* and b* values. The color of wine chroma decreases continuously during aging, and the hue shifts from red to orange [44]. This suggests that the levels of flavan*-3-*ols in red wine are closely linked to the color expression of anthocyanin derivatives during fermentation and aging.

## 5. Conclusions

This study investigated the evolution and correlation of phenolic substances and color characteristics in ‘Merlot’ and ‘Marselan’ dry red wines, throughout the entire process, including cold maceration, alcoholic fermentation, malolactic fermentation, and one-year bottle storage. This study shows that the leaching of phenolic compounds from grapes is limited during cold maceration. However, there is a primary rapid release during alcohol fermentation, which leads to the production of pyranoanthocyanins. Polymeric pigments are formed and increase during the latter stage of alcohol fermentation, remaining at a high level from the malolactic fermentation through storage. The leaching and evolution patterns of most phenolic compounds in the two varieties are found to be comparable. The red-purple color of ‘Merlot’ and ‘Marselan’ wines during the aging process is primarily determined by the concentrations of anthocyanins and F-A polymeric pigments ((E)cat-Mv, (E)gcat-Mv, (E)cat-MvA, (E)cat-MvC). Furthermore, the red color of ‘Merlot’ wine is strongly associated with the concentrations of pinotins. In both wine varieties, the concentrations of four flavan*-3-*ol components have a strong negative correlation with A-e-F, and A-v-F pigments, as well as the color indices *h_ab_* and b* values. Conversely, there is a positive correlation with F-A (A-F), pinotins, monomeric anthocyanins, and the color indices a* and C*_ab_ values.

Based on these findings, it is believed that the red-purple components may vary slightly between the two wine varieties. Increasing the level of flavan*-3-*ols in wine may improve the stability of the red-purple hue, potentially solving the issue of unstable color and rapid fading in red wine produced in the eastern foothills of the Helan Mountains in Ningxia.

## Figures and Tables

**Table 1:** Concentrations of phenolic compounds in the skins of ‘Merlot’ and ‘Marselan’ grapes (mg/kg).

Category	Phenolic Compounds	‘Merlot’	‘Marselan’
Anthocyanins	Cyanidin*-3-O-*glucoside	28.18 ± 7.67 *	9.18 ± 3.39
	Cyanidin*-3-O-*acetylglucoside	4.89 ± 1.32 *	1.1 ± 0.27
	Cyanidin*-3-O-*coumarylglucoside	3.35 ± 1.18 *	0.87 ± 0.38
	Peonidin*-3-O-*glucoside	79.96 ± 17.36 *	37.8 ± 7.37
	Peonidin*-3-O-*acetylglucoside	16.78 ± 1.47	22.72 ± 1.38 *
	Peonidin*-3-O-*coumarylglucoside	25.1 ± 3.34 *	15.98 ± 0.97
	Delphinidin*-3-O-*glucoside	44.85 ± 7.62	65.8 ± 6.46 *
	Delphinidin*-3-O-*acetylglucoside	8.97 ± 1.36	10.03 ± 1.31
	Delphinidin*-3-O-*coumarylglucoside	1.86 ± 0.55	6.06 ± 0.39 *
	Petunidin*-3-O-*glucoside	36.46 ± 4.56	62.86 ± 3.42 *
	Petunidin*-3-O-*acetylglucoside	29.23 ± 3.52 *	13.01 ± 2.01
	Petunidin*-3-O-*coumarylglucoside	4.54 ± 0.7	13.77 ± 0.75 *
	Malvidin*-3-O-*glucoside	99.07 ± 0.63	237.6 ± 10.24 *
	Malvidin*-3-O-*acetylglucoside	69.5 ± 5.2	135.21 ± 8.77 *
	Malvidin*-3-O-*coumarylglucoside	29.19 ± 1.09	99.04 ± 8.41 *
Flavonols	Kaempferol-galactoside	7.55 ± 0.83	16.1 ± 2.4 *
	Kaempferol-glucoside	3.46 ± 0.38	4.88 ± 0.35 *
	Quercetin-galactoside	30.79 ± 5.62 *	19.7 ± 2.49
	Quercetin-glucoside	43.51 ± 1.8	48.71 ± 2.73
	Quercetin-glucuronide	37.06 ± 1.8	74.01 ± 8.95 *
	Isorhamnetin-glucoside	8.46 ± 0.73	18.01 ± 1.43 *
	Myricetin-galactoside	14.75 ± 0.93 *	11.11 ± 0.81
	Myricetin-glucoside	9.23 ± 0.32	9.46 ± 0.47
	Syringetin-glucoside	26.98 ± 2.21 *	10.27 ± 0.56
Flavan*-3-*ols	Epicatechin-free unit	0.57 ± 0.01	0.7 ± 0.01 *
	Catechin-end unit	36.74 ± 5.96 *	27.15 ± 2.18
	Epicatechin-end unit	5.24 ± 0.63 *	3.51 ± 0.61
	Epigallocatechin-end unit	15.26 ± 2.45 *	6.23 ± 0.36
	Epicatechin gallate-end unit	1.34 ± 0.25	1.43 ± 0.3
	Catechin-extension unit	821.53 ± 52.57	1473.51 ± 54.93 *
	Epicatechin-extension unit	24.02 ± 1.15	28.65 ± 1.12 *
	Epigallocatechin-extension unit	134.09 ± 4.56	160.71 ± 2.86 *
	Epicatechin gallate-extension unit	1.67 ± 0.16	4.15 ± 0.41 *
	Mean degree of polymerization	13.05 ± 1.24	32.19 ± 3.37 *

“Mean ± sd” (*n* = 3) are shown in the table. Asterisk * represents significant differences in the concentrations of different phenolic compounds between different grape varieties at *p* ≤ 0.05.

**Table 2:** Physicochemical indices of musts and wines.

Samples	Parameters	‘Merlot’	‘Marselan’
Juice	Total soluble solid (°Brix)	25.7 ± 0.1	26.2 ± 0.2 *
	Titratable acidity (g/L)	4.2 ± 0.06	4.7 ± 0.04 *
	pH	3.67 ± 0.01 *	3.21 ± 0.02
Wine	Residual sugar (g/L)	3.63 ± 0.29	4.87 ± 0.31 *
	Total acid (g/L)	6.80 ± 0.01	7.80 ± 0.01 *
	Volatile acid (g/L)	0.67 ± 0.01	0.67 ± 0.02
	Ethanol (%, *v*/*v*)	14.31 ± 0.01	14.88 ± 0.04 *
	Free SO2/(mg/L)	2.57 ± 0.03	3.50 ± 0.06 *
	pH	3.54 ± 0.01 *	3.28 ± 0.01

“Mean ± sd” (*n* = 3) were shown in the table. Asterisk * represents significant differences between different grape varieties at *p* ≤ 0.05.

**Table 3:** Total concentrations (mg/L) of phenolic compounds in various categories in ‘Merlot’ and ‘Marselan’ wines at the end of malolactic fermentation.

Category	Phenolic Compounds	‘Merlot’		‘Marselan’	
		Amount of Components	Concentration of Each Category	Amount of Components	Concentration of Each Category
Anthocyanins	Cyanidin	3	8.53 ± 0.50 *	3	6.19 ± 0.31
	Peonidin	3	50.75 ± 3.54 *	3	25.68 ± 1.18
	Delphinidin	3	22.16 ± 1.25	3	25.02 ± 1.69 *
	Petunidin	3	28.33 ± 1.85	3	29.48 ± 1.57
	Malvidin	3	160.27 ± 9.49	3	157.09 ± 2.52
Pyranoanthocyanins	Vitisin A	3	2.19 ± 0.20	3	3.68 ± 0.31 *
	Vitisin B	3	7.25 ± 0.46 *	3	4.47 ± 0.17
	Pinotin	4	3.72 ± 0.12 *	4	1.93 ± 0.19
Polymeric pigments	F-A (A-F)	2	0.54 ± 0.08	4	2.16 ± 0.22 *
	A-e-F	4	4.30 ± 0.49	4	5.03 ± 0.50
Non-anthocyanin phenols	Flavonol	10	54.30 ± 3.62 *	10	34.35 ± 4.23
	Favan*-3-*ol	5	105.81 ± 9.12 *	5	66.32 ± 9.02
	Phenolic acid	9	95.73 ± 8.10 *	11	76.67 ± 5.51

“Mean ± sd” (*n* = 3) were shown in the table. Asterisk * represents significant differences in the concentrations of different phenolic compounds between different grape varieties at *p* ≤ 0.05.

## Data Availability

Data is contained within the article or Supplementary Materials.

## Supplementary Materials

The following supporting information can be downloaded at: https://www.mdpi.com/article/10.3390/foods13030494/s1.

## Abbreviations

Abbreviations and their full names in this article.

A-e-F	Anthocyanin ethyl-linked flavanols bridged polymerized pigments
A-F	Direct anthocyanin-flavanol polymerized pigments (A type)
ANOVA	Analysis of variance
A-v-F	Flavanyl-pyranoanthocyanins
BAF	Before alcohol fermentation
BC	Before cooling
BH	Before heating
BMLF	Before malolactic fermentation
Cy-ac	Cyanidin*-3-O-*acetylglucoside
Cy-co	Cyanidin*-3-O-*coumarylglucoside
Cy-Glu	Cyanidin*-3-O-*glucoside
Di(e)cat-Mv	Di(epi)catechin-malvidin*-3-O-*glucoside (B type)
Dp-ac	Delphinidin*-3-O-*acetylglucoside
Dp-co	Delphinidin*-3-O-*coumarylglucoside
Dp-Glu	Delphinidin*-3-O-*glucoside
EAF	End of alcohol fermentation
EC	End of cooling
(E)cat-Mv	(Epi)catechin-malvidin*-3-O-*Glucoside (B type)
(E)cat-MvA	(Epi)catechin-malvidin*-3-O-*acetylglucoside (B type)
(E)cat-MvC	(Epi)catechin-malvidin*-3-O-*coumaroylglucoside (B type)
(E)gcat-Mv	(Epi)gallocatechin-malvidin*-3-O-*glucoside (B type)
EH	End of heating
EMLF	End of malolactic fermentation
F-A	Direct flavanol-anthocyanin polymerized pigments (B type)
HPLC-QqQ	High-performance liquid chromatography tandem triple-quadrupole
MvA-aldehyde	Malvidin*-3-O-*acetylglucoside-acetaldehyde
Mv-ac	Malvidin*-3-O-*acetylglucoside
MvA-e-(e)cat	Malvidin*-3-O-*acetylglucoside-ethyl-(epi)cate
Mv-aldehyde	Malvidin*-3-O-*glucoside-acetaldehyde
MvA-py acid	Malvidin*-3-O-*acetylglucoside-pyruvic acid
MvA-vcol	Malvidin*-3-O-*acetylglucoside*-4-*vinylcatechol
MvA-v(e)cat	Malvidin*-3-O-*acetylglucoside*-4-*vinyl(epi)catechin
MvA-vgol	Malvidin*-3-O-*acetylglucoside*-4-*vinylguaiacol
MvA-vpol	Malvidin*-3-O-*acetylglucoside*-4-*vinylphenol
MvC-aldehyde	Malvidin*-3-O-*coumaroylglucoside-acetaldehyde
MvC-e-(e)cat	Malvidin*-3-O-*coumaroylglucoside-ethyl-(epi)catechin
Mv-co	Malvidin*-3-O-*coumarylglucoside
MvC-py acid	Malvidin*-3-O-*coumaroylglucoside-pyruvic acid
MvC-v(e)cat	Malvidin*-3-O-*coumaroylglucoside*-4-*vinyl(epi)catechin
MvC-vgol	Malvidin*-3-O-*coumaroylglucoside*-4-*vinylguaiacol
MvC-vphol	Malvidin*-3-O-*coumaroylglucoside*-4-*vinylphenol
Mv-(e)cat	Malvidin*-3-O-*glucoside-(epi)catechin (A type)
Mv-e-di(e)cat	Malvidin*-3-O-*glucoside-ethyl-di(epi)catechin
Mv-e-(e)cat	Malvidin*-3-O-*glucoside-ethyl—(epi)catechin
Mv-Glu	Malvidin*-3-O-*glucoside
Mv-p-vphol	Malvidin*-3-O-*glucoside-pyrano-vinylphenol
Mv-py acid	Malvidin*-3-O-*glucoside-pyruvic acid
Mv-vcol	Malvidin*-3-O-*glucoside*-4-*vinylcatechol
Mv-vdi(e)cat	Malvidin*-3-O-*glucoside*-4*-vinyl-di(epi)catechin
Mv-v(e)cat	Malvidin*-3-O-*glucoside*-4*-vinyl(epi)catechin
Mv-vgol	Malvidin*-3-O-*glucoside*-4*-vinylguaiacol
Mv-vpol	Malvidin*-3-O-*glucoside*-4*-vinylphenol
Pe-ac	Peonidin*-3-O-*acetylglucoside
Pe-co	Peonidin*-3-O-*coumarylglucoside
Pe-Glu	Peonidin*-3-O-*glucoside
PLSR	Partial Least Squares Regression Analysis
Pt-ac	Petunidin*-3-O-*acetylglucoside
Pt-co	Petunidin*-3-O-*coumarylglucoside
Pt-Glu	Petunidin*-3-O-*glucoside
SGto1	Specific gravity reduced to 1
3M	Storage for 3 months
6M	Storage for 6 months
9M	Storage for 9 months
12M	Storage for 12 months
